# Health Benefits of Probiotics: A Review

**DOI:** 10.5402/2013/481651

**Published:** 2013-01-02

**Authors:** Maria Kechagia, Dimitrios Basoulis, Stavroula Konstantopoulou, Dimitra Dimitriadi, Konstantina Gyftopoulou, Nikoletta Skarmoutsou, Eleni Maria Fakiri

**Affiliations:** ^1^Microbiology Department, Sismanoglion General Hospital of Athens, 15126 Athens, Greece; ^2^Internal Medicine Department, APPK, Laiko Hospital of Athens, 11527 Athens, Greece

## Abstract

Probiotic bacteria have become increasingly popular during the last two decades as a result of the continuously expanding scientific evidence pointing to their beneficial effects on human health. As a result they have been applied as various products with the food industry having been very active in studying and promoting them. Within this market the probiotics have been incorporated in various products, mainly fermented dairy foods. In light of this ongoing trend and despite the strong scientific evidence associating these microorganisms to various health benefits, further research is needed in order to establish them and evaluate their safety as well as their nutritional aspects. The purpose of this paper is to review the current documentation on the concept and the possible beneficial properties of probiotic bacteria in the literature, focusing on those available in food.

## 1. Introduction

The association of probiotics with well-being has a long history. More than a century has passed since Tissier observed that gut microbiota from healthy breast fed infants were dominated by rods with a bifid shape (bifidobacteria) which were absent from formula fed infants suffering from diarrhoea, establishing the concept that they played a role in maintaining health. Since then a series of studies have supported this association but they were originally poorly designed and controlled and faced practical challenges such as strain specificity of properties and the slow growth of probiotics in substrates other than human milk. By time, they have successfully evolved with the more recent ones accumulating more substantial evidence that probiotic bacteria can contribute to human health. These data have coincided with the increasing consumer awareness about the relationship between health and nutrition creating a supporting environment for the development of the functional food concept introduced to describe foods or food ingredients exhibiting beneficial effects on the consumers' health beyond their nutritive value. The functional food market is expanding, especially in Japan—its birthplace—with further growth prospects in Europe and the United States and in most countries the largest share of its products is held by probiotics [1, 2]. The reported beneficial effects of probiotic consumption include improvement of intestinal health, amelioration of symptoms of lactose intolerance, and reduction of the risk of various other diseases, and several well-characterized strains of *Lactobacilli* and *Bifidobacteria* are available for human use [3, 4]. Nevertheless, despite the promising evidence, the role of probiotics in human health as well as the safety of their application should be further investigated as the current knowledge of the characteristics that are necessary for their functionality in the gut is not complete. 

## 2. Definition of Probiotics and Other Related Terms

Etymologically the term probiotic is derived from the Greek language meaning “for life” but the definition of probiotics has evolved over time simultaneously with the increasing interest in the use of viable bacterial supplements and in relation to the progress made in understanding their mechanisms of action. The term was originally used to describe substances produced by one microorganism that stimulated the growth of others and was later used to describe tissue extracts that stimulated microbial growth and animal feed supplements exerting a beneficial effect on animals by contributing to their intestinal flora balance [5]. Until recently the most widely used definition which contributed to the development of the probiotic concept in several ways was that of Fuller: “probiotics are live microbial feed supplements which beneficially affect the host animal by improving microbial balance” [6]. The definition used at present was given by the Food and Agriculture Organization of the United Nations World Health Organization, according to which probiotics are redefined as “live microorganisms which when administered in adequate amounts confer a health benefit on the host.” In relation to food the definition can be adjusted by emphasizing that the beneficial effect is exerted by the microorganisms “when consumed in adequate amounts as part of food” [7].

The term prebiotics was introduced by Gibson and Roberfroid in 1995 to describe food supplements that are nondigestible by the host but are able to exert beneficial effects by selective stimulation of growth or activity of microorganisms that are present in the intestine. Prebiotic substances are not hydrolysed nor absorbed in the gastrointestinal tract but are available as substrates for probiotics and the most commonly used ones at present are nondigestible fructooligosaccharides. For practical reasons the combination of probiotics and prebiotics has been described as conbiotics by certain authors and as symbiotics by others [8, 9]. Although prebiotics seem to have a role in health promotion this needs to be supported by further studies. During the last years the concept of functional food has also been developed in order to describe foods containing ingredients with positive effects on host health beyond their nutritive value. They include those products that contain biologically active components that improve health, such as probiotics [1]. 

## 3. Microbial Species with Applications as Probiotics

Taking into consideration their definition the number of microbial species which may exert probiotic properties is impressive. Some of the most important representatives are listed in Table 1. As far as nutrition is concerned only the strains classified as lactic acid bacteria are of significance and among them the ones with the most important properties in an applied context are those belonging to the genera *Lactococcus* and *Bifidobacterium* [10]. Lactic acid bacteria are Gram-positive, catalase-negative bacterial species able to produce lactic acid as main end-product of carbohydrate fermentation. The genus *Bifidobacterium* is therefore rather traditionally than phylogenetically listed among them as they use a separate metabolic pathway. Two other species playing an important role in the food industry, particularly dairy products, although not strictly considered as probiotics are *Streptococcus thermophilus *and *Lactococcus lactis*, two of the most commercially important lactic acid bacteria [11].

It is important to mention that since probiotic activities are strain related, strain identification is recommended in order to establish their suitability and performance for industrial application. This is achieved by a combination of phenotypic tests followed by genetic identification using molecular techniques like DNA/DNA hybridisation, 16SRNA sequencing, and so forth [7].

## 4. Desirable Probiotic Properties

In order for a potential probiotic strain to be able to exert its beneficial effects, it is expected to exhibit certain desirable properties. The ones currently determined by in vitro tests are

- acid and bile tolerance which seems to be crucial for oral administration,
- adhesion to mucosal and epithelial surfaces, an important property for successful immune modulation, competitive exclusion of pathogens, as well as prevention of pathogen adhesion and colonisation,
- antimicrobial activity against pathogenic bacteria,
- bile salt hydrolase activity.

Nevertheless, the value of these parameters is still under debate as there are matters of relevance, in vivo and in vitro discrepancies, and lack of standardization of operating procedures to be considered. As there are no specific parameters essential to all probiotic applications, the best approach to establish a strain's properties is target population and target physiologic function specific studies [12–14].

As far as the final product is concerned, the probiotic dose levels should be based on the ones found to be efficacious in human studies and the colony forming units per gram of product is an important parameter. Although the information about the minimum effective concentrations is still insufficient, it is generally accepted that probiotic products should have a minimum concentration of 10^6^ CFU/mL or gram and that a total of some 10^8^ to 10^9^ probiotic microorganisms should be consumed daily for the probiotic effect to be transferred to the consumer. Furthermore, the strains must be able to grow under manufacture and commercial conditions and should retain viability under normal storage conditions [3, 15]. Viability is by definition a prerequisite for probiotic functionality as it potentiates mechanisms such as adherence, reduction of gut permeability, and immunomodulation and constitutes an industrial challenge [16, 17]. Nevertheless, certain studies have demonstrated that viability is not necessary for all probiotic effects as not all mechanisms nor clinical benefits are directly related to viability and that even cell wall components on some probiotic bacteria or probiotic DNA may have significant health effects. Thus for certain probiotic strains optimal growth during the initial production steps might be sufficient and they may not need to retain good viability during storage [18, 19].

## 5. Mechanisms of Probiotic Activity

Probiotics have various mechanisms of action although the exact manner in which they exert their effects is still not fully elucidated. These range from bacteriocin and short chain fatty acid production, lowering of gut pH, and nutrient competition to stimulation of mucosal barrier function and immunomodulation. The latter in particular has been the subject of numerous studies and there is considerable evidence that probiotics influence several aspects of the acquired and innate immune response by inducing phagocytosis and IgA secretion, modifying T-cell responses, enhancing Th1 responses, and attenuating Th2 responses [20–22].

## 6. Probiotics and Food Products

The range of food products containing probiotic strains is wide and still growing. The main products existing in the market are dairy-based ones including fermented milks, cheese, ice cream, buttermilk, milk powder, and yogurts, the latter accounting for the largest share of sales [23, 24].

 Nondairy food applications include soy based products, nutrition bars, cereals, and a variety of juices as appropriate means of probiotic delivery to the consumer [25, 26]. The factors that must be addressed in evaluating the effectiveness of the incorporation of the probiotic strains into such products are, besides safety, the compatibility of the product with the microorganism and the maintenance of its viability through food processing, packaging, and storage conditions. The product's pH for instance is a significant factor determining the incorporated probiotic's survival and growth, and this is one of the reasons why soft cheeses seem to have a number of advantages over yoghurt as delivery systems for viable probiotics to the gastrointestinal tract [27–29]. Current technological innovations provide ways to overcome probiotic stability and viability issues offering new options for their incorporation in new media and subsequent satisfaction of the increasing consumer demand. Microencapsulation technologies have been developed to protect the bacteria from damage caused by external environment. By the introduction of a straw delivery system containing a dry form of the probiotic bacterium beverage manufacturers can now provide it to the consumer. In addition, viable spores of a spore forming probiotic are available in the market offering advantages during processing. In the same time, the potential of lantibiotics'—substances with antimicrobial properties—production by bifidobacteria is being explored in order to be applied in the food area [30, 31].

## 7. Health Benefits of Probiotics

There is increasing evidence in favour of the claims of beneficial effects attributed to probiotics, including improvement of intestinal health, enhancement of the immune response, reduction of serum cholesterol, and cancer prevention. These health properties are strain specific and are impacted by the various mechanisms mentioned above. While some of the health benefits are well documented others require additional studies in order to be established. In fact, there is substantial evidence to support probiotic use in the treatment of acute diarrhoeal diseases, prevention of antibiotic-associated diarrhoea, and improvement of lactose metabolism, but there is insufficient evidence to recommend them for use in other clinical conditions.

## 8. Antibiotic-Associated Diarrhoea

Mild or severe episodes of diarrhoea are common side effects of antibiotic therapy as the normal microflora tends to be suppressed, encouraging the overgrowth of opportunistic or pathogenic strains. The spectrum may range from diarrhoea without mucosal abnormality to pseudomembranous colitis. The latter is a severe form of antibiotic-associated diarrhoea (caused by *Clostridium difficile*, cytotoxic strains of which may emerge after antibiotic use). The name of the condition is derived from the plaque-like adhesion of fibrinopurulent material to the damaged mucosal layer and it is characterized by diarrhoea, abdominal distention, vomiting, fever, and leukocytosis and if untreated might lead to complications such as toxic megacolon and perforation. Treatment consists of withdrawal of the causal antibiotic agent, correction of the electrolyte disorders, and in severe cases therapy with metronidazole or vancomycin. Treatment with probiotics has been used in clinical practice with *L. rhamnosus *and* S. boulardii *being administered. Several studies that have been carried out suggest that probiotic use is associated with a reduced risk of antibiotic-associated diarrhoea [32, 33]. A recent meta-analysis evaluating the available evidence on probiotics for the prevention and treatment of antibiotic-associated diarrhoea concluded that probiotic administration- (namely, *L. rhamnosus, L. casei*, and the yeast *S. boulardii*, as these are the probiotics predominantly included in the majority of trials) is associated with a reduced risk of the condition. Matters for future research include the optimal dose of the probiotic preparation and the comparative effectiveness of different probiotic interventions [34].

## 9. Infectious Diarrhoea 

Treatment and prevention of infectious diarrhoea are probably the most widely accepted health benefits of probiotic microorganisms. Rotavirus is the most common cause of acute infantile diarrhoea in the world and a significant cause of infant mortality. The virus replicates in the highly differentiated absorptive columnar cells of the small intestinal epithelium and the normal microflora seems to play an important role in the host response to the infection, as it has been shown that absorption of antigens is more enhanced in germ-free than in normal mice [35]. Probiotic supplementation of infant formulas has been aimed both at the prevention of rotaviral infections and the treatment of established disease. Well-controlled clinical studies have shown that probiotics such as *L. rhamnosus* GG, *L. reuteri*, *L. casei* Shirota, and *B. animalis* Bb12 can shorten the duration of acute rotavirus diarrhoea with the strongest evidence pointing to the effectiveness of *L. rhamnosus* GG and *B. animalis* Bb12 [36–38]. The proposed mechanisms include competitive blockage of receptor site signals regulating secretory and motility defences, enhancement of the immune response, and production of substances that directly inactivate the viral particles. In addition to rotavirus infection there is evidence that certain food as well as nonfood probiotic strains can inhibit the growth and adhesion of a range of diarrhoeal syndromes. The benefit of probiotics such as *L. reuteri*, *L. rhamnosus *GG, *L. casei*, and *S. boulardii* in reducing the duration of acute diarrhoea in children has been demonstrated, [37, 39]. For example, in a prospective, randomized, controlled French study conducted among children in day care, the administered probiotic yoghurt product containing *L. casei *shortened the mean duration of diarrhoea significantly compared to the conventional one [40]. Several studies have investigated the efficacy of probiotics in the prevention of travellers' diarrhoea in adults. Although results are quite contradictory, due to differences in study populations, type of probiotic being investigated, applied doses, as well as trip destination and traveller compliance, *L. rhamnosus* GG, *S. boulardii*, *L. acidophilus,* and *B. bifidum* seem to exhibit significant efficacy [41–43]. Furthermore, numerous animal studies have indicated an inhibitory effect of probiotics against enteropathogens mainly through the production of bacteriocins [44].

## 10. Lactose Intolerance

Lactose intolerance is a genetically determined beta-galactosidase deficiency resulting in the inability to hydrolyse lactose into the monosaccharides glucose and galactose. Upon reaching the large bowel the undigested lactose is degraded by bacterial enzymes leading to osmotic diarrhoea. Acquired, usually reversible, causes of beta-galactosidase deficiency include pelvic radiotherapy which damages the mucosa, as well as infection with rotavirus which infects lactase producing cells, and short bowel syndrome. Lactose intolerant individuals develop diarrhoea, abdominal discomfort, and flatulence after consumption of milk or milk products. Although conventional yoghurt preparations, using *S. thermophilus* and *L. delbrueckii* ssp. *Bulgaricus,* are even more effective in this direction, partly because of higher beta-galactosidase activity, improvement of lactose metabolism is a claimed health benefit attributed to probiotics and seems to involve certain strains more than others and in specific concentrations. Therefore and as certain individuals have responded positively to probiotic supplementation, clinicians should consider it as a therapeutic alternative [45, 46].

## 11. Probiotics and Allergy

Recent evidence suggests that exposure to bacteria in early life may exhibit a protective role against allergy and in this context probiotics may provide safe alternative microbial stimulation needed for the developing immune system in infants. In the same time they improve mucosal barrier function, a property that is considered to contribute in moderating allergic response. The role of intestinal microbiota in allergy is supported by observations of their quantitative as well as qualitative differences among children and infants suffering from allergies and healthy ones, the former exhibiting colonization by a more adult-like type of microflora [42, 47–49]. These probiotic effects seem to particularly involve food allergy and atopic dermatitis. The latter is a common chronic relapsing skin disorder of infancy and childhood with hereditary predisposition being an important component of its pathogenesis together with the individual's exposure to environmental allergens. A limited number of strains have been tested for their efficacy in the treatment and prevention of allergy in infants. In a recent study of breast fed infants suffering from atopic eczema *B. lactis* and *L. rhamnosus* GG were found to be effective in decreasing the eczema severity. Furthermore *L. rhamnosus *GG has been found successful in preventing the occurrence of atopic eczema in high risk infants, when supplied prenatally to selected mothers who had at least one first degree relative with atopic eczema, allergic rhinitis, or asthma [50]. Probiotics however have not been very successful in alleviating symptoms of asthma [51]. As far as food allergy is concerned, it is described as an immunologically mediated adverse reaction against dietary antigens leading to secondary intestinal inflammation and disturbances. The mechanisms of the immune modulating effect of *L. rhamnosus *GG are not entirely understood but seem be related to the antigen's transport across the intestinal mucosa [52]. Recently the use of probiotic preparations in adults with milk hypersensitivity—not lactose intolerance—has been studied, concluding that certain strains may suppress the milk-induced inflammatory response and improve allergy symptoms; nevertheless further study in the field is necessary [53, 54].

## 12. Other Health Benefits

The list of health benefits mediated by probiotics is not limited to the ones mentioned so far and includes a range of promising effects that require however further human studies in order to be substantiated. There is evidence that probiotic bacteria are dietary components that may play a role in decreasing cancer incidence. The exact mechanisms are under investigation, but studies have demonstrated that certain members of *Lactobacillus* and *Bifidobacterium* spp. decrease the levels of carcinogenetic enzymes produced by colonic flora through normalization of intestinal permeability and microflora balance as well as production of antimutagenic organic acids and enhancement of the host's immune system [55, 56]. Furthermore, evidence suggests that food products containing probiotic bacteria could possibly contribute to coronary heart disease prevention by reducing serum cholesterol levels as well as to blood pressure control. Proposed mechanisms include interference with cholesterol absorption from the gut, direct cholesterol assimilation, and production of end fermentation products that affect the systemic levels of blood lipids and mediate an antihypertensive effect. Nevertheless, these probiotic effects are still a matter of debate as further research is needed in long-term human studies [57]. Last but not least, probiotic strains administered in dairy products have shown to improve the therapeutic outcome in women with bacterial vaginosis, most probably by supporting the normal vaginal lactobacilli microbiota [58].

## 13. Conclusions

There is scientific evidence supporting the incorporation of probiotics in nutrition as a means of derivation of health benefits. This evidence seems adequate concerning the prevention and treatment of certain conditions while simply promising or even controversial when it comes to others. The best documented effects include bowel disorders such as lactose intolerance, antibiotic-associated diarrhoea and infectious diarrhoea, and allergy, and emerging evidence accumulates concerning their potential role in various other conditions. In the same time as relevant consumer awareness grows, such products are becoming increasingly popular and tend to represent one of the largest functional food markets. Dairy products, particularly yoghurt, continue to be the most important vehicles for delivery of probiotic bacteria to the consumer with the nondairy sector continuously evolving as well, as a result of food technology advances and the growing demand. A virtuous circle is therefore created: as the range of new products with improved sensory appeal widens, consumer acceptance increases and the food industry invests more on this growing market by development of new processes and products. Nevertheless, the development of probiotics for human consumption is still in its infancy. Further research, in the form of controlled human studies, is needed to determine which probiotics and which dosages are associated with the greatest efficacy and for which patients, as well as to demonstrate their safety and limitations. In addition, the regulatory status of probiotics as food components needs to be established on an international level with emphasis on efficacy, safety, and validation of health claims on food labels. There is no doubt that we will witness a significant increase in the role of probiotics in nutrition and medicine over the next decade and while their application in the prevention and treatment of various disorders should be considered by medical professionals and promoted by the food industry, this should be done with skepticism and respect to the consumer.

## Figures and Tables

**Table 1:** Adapted from Holzapfel et al., 2001 [10].

Microorganisms considered as probiotics	
*Lactobacillus* species	*Bifidobacterium* species
*L. acidophilus* *L. casei* *L. crispatus* *L. gallinarum* ^1^ *L. gasseri* *L. johnsonii* *L. paracasei* *L. plantarum* *L. reuteri* *L. rhamnosus *	*B. adolescentis* *B. animalis* *B. bifidum* *B. breve* *B. infantis* *B. lactis* ^2^ *B. longum *

Other lactic acid bacteria	Nonlactic acid bacteria

*Enterococcus f* *ae* *ca* *li* *s* ^1^ *E. faecium* *Lactococcus lactis* ^3^ *Leuconostoc mesenteroides* *Pediococcus acidilactici* ^3^ *Sporolactobacillus inulinus* ^1^ *Streptococcus thermophilus* ^3^	*Bacillus cereus var. *toyo*i* ^1^ *Escherichia coli * strain nissle *Propionibacterium freudenreichii* *Saccharomyces cerevisiae* *S. boulardii *

^1^Mainly used for animals.

^2^Recently reclassified as *B. animalis *subsp. *lactis* [59].

^3^Little is known about probiotic properties.
